# Perinatal anxiety disorders screening study: a study protocol

**DOI:** 10.1186/s12888-024-05575-9

**Published:** 2024-02-24

**Authors:** Nichole Fairbrother, Bryn Stagg, Olivia Scoten, Cora Keeney, Claudia Cargnelli

**Affiliations:** 1 Department of Family Practice, University of British Columbia, Vancouver, Canada; 2 Department of Obstetrics and Gynaecology, University of British Columbia, Vancouver, Canada; 3 Faculty of Medicine, University of British Columbia, Vancouver, Canada

**Keywords:** Anxiety disorders, Perinatal anxiety, Screening, Assessment, Pregnancy, Postpartum

## Abstract

**Background:**

The anxiety and their related disorders (AD) are the most common of all mental health conditions, and affect approximately 20% of pregnant and postpartum people. They are associated with significant distress and life interference for sufferers, as well as negative consequences for fetal and infant development. At present, little if any routine screening for prenatal AD is being conducted and data regarding the most effective tools to screen for these disorders is lacking. The majority of screening studies suffer from methodological difficulties which undermine the confidence needed to recommend measures for population distribution. The primary purpose of this research is to identify the most accurate self-report tool(s) to screen for perinatal AD.

**Methods:**

A large, prospective cohort of pregnant people (*N* = 1,000) is being recruited proportionally across health service delivery regions in British Columbia (BC). The screening accuracy of a broad range of perinatal AD self-report measures are being assessed using gold standard methodology. Consenting individuals are administered online questionnaires followed by a semi-structured diagnostic interview between 16- and 36-weeks’ gestation, and again between 6 and 20 weeks postpartum. Questionnaires include all screening measures, measures of sleep and unpaid family work, and questions pertaining to demographic and reproductive history, COVID-19, gender role burden, and mental health treatment utilization. Interviews assess all current anxiety disorders, as well as obsessive–compulsive disorder, and posttraumatic stress disorder.

**Discussion:**

This research is in response to an urgent demand for accurate perinatal AD screening tools based on high quality evidence. AD among perinatal people often go unidentified and untreated, resulting in continued suffering and life impairment. Findings from this research will inform healthcare providers, policymakers, and scientists, about the most effective approach to screening for anxiety and related disorders in pregnancy in the postpartum period.

## Introduction

For many health conditions, screening is the first step in a pathway to effective, and in some cases lifesaving, interventions [1–3]. It allows for population level early disease detection thereby identifying those in need of further assessment and ultimately treatment. Further, screening provides significant cost savings [4–8] by ensuring that only those who require more time and labour-intensive diagnostic assessments are offered them [4, 8–10]. Although screening for mental health conditions has been shown to lead to improved mental health even in the absence of a clear pathway to treatment [11, 12], there are still several gaps in screening for mental health conditions in Canada, particularly in the perinatal period [13].

The current edition of the Diagnostic and Statistical Manual of Mental Disorders (DSM-5) [14] includes ten anxiety disorders and several anxiety-related disorders (AD). For the purposes of this research, we have defined AD to include all DSM-5 anxiety disorders as well as two anxiety-related disorders: posttraumatic stress disorder (PTSD) and obsessive–compulsive disorder (OCD) [14]. The AD affect one in five pregnant and postpartum people [15], and are of high importance for perinatal people both due to the fact that they are highly prevalent, but also because they are associated with a range of negative reproductive health outcomes [15–17]. These include: obstetrical (e.g., miscarriage, low birth weight, preterm delivery) [16, 18–21]; fetal and infant (e.g., impaired self-regulation and motor development, and an increased risk for attention-deficit/hyperactivity disorder) [16, 22–28]; and the birthing parent (e.g., postpartum depression, reduced employment capacity, unemployment, and decreased social, emotional and physical functioning) [16, 17, 29–35]. The AD are also associated with significant health care costs and increased health care service utilization [36–39].

Routine screening for perinatal depression is employed in many parts of the world [40–42]. This has been made possible through the availability of screening tools for perinatal depression of high and well-established accuracy [43, 44]. Despite the fact that, among perinatal people, AD are significantly more common that perinatal depression (21% for perinatal AD, and approximately 6% for perinatal depression) [15, 45] screening for these conditions is almost non-existent [4]. This is largely the result of a lack of identifiable screening tools with established and high accuracy [46]. Numerous self-report screening tools for perinatal AD have been developed and to some extent evaluated [47]. More than one may, in fact, prove to be highly accurate. However, assessments of their accuracy have been burdened by methodological weaknesses of sufficient magnitude such that, at present, no screening tool for perinatal AD can be recommended with confidence [48].

What makes the establishment of broadly disseminated screening for perinatal AD so appealing is the following. First, perinatal people are highly accepting of screening programs, but tend not to discuss their mental health difficulties unless asked about them [4, 49]. Consequently, without screening, perinatal AD will often go undetected [50]. Second is the recent increase in the availability and acceptability of highly effective, evidence-based treatments for perinatal AD. Specifically, cognitive behaviour therapy (CBT), a form of talk therapy, is the treatment of choice for many anxiety-related conditions. Randomized controlled trials comparing CBT with medication approaches have found that CBT is safe and equal or superior to medication [51–53], and a recent systematic review found CBT to effective for perinatal populations [54]. Historically, access to CBT has been limited and not broadly publicly funded due to the fact that it is time-consuming and expensive [55–57]. More recently, however, CBT has become increasingly available in self-administered and online formats, greatly improving accessibility [58, 59], without any loss in effectiveness [60, 61]. Accordingly, when screening leads to appropriate treatment, positive outcomes are highly likely.

The need for accurate, evidence-based screening tools is now more critical in light of recent urgent calls by various healthcare agencies for perinatal AD screening, and guidance regarding screening tool selection. These include the Perinatal Services BC [62], the American College of Obstetricians and Gynecologists [63], MGH Centre for Women’s Mental Health [64].

As population-wide administration of screening programs is a large, expensive undertaking, and the primary determinant of who will receive more costly diagnostic assessments, ensuring that resources are well spent is critical and can only be achieved when valid screening tools are employed. To be valid, screening tools must demonstrate a high level of accuracy when compared with gold standard diagnostic assessments. For this to occur, very specific research methodology is required. The Cochrane Screening & Diagnostic Tests Methods Group recommends the Quality of Diagnostic Accuracy Studies (QUADAS-2) [48, 65] as the criteria of choice for assessing studies of diagnostic accuracy [65–67]. QUADAS-2 criteria were also formally accepted by the Australian National Health and Medical Research Councils [4]. The QUADAS-2 criteria are outlined below, with some language adjusted to reflect requirements when screening for mental health difficulties among perinatal people. Specifically, assessments of perinatal AD screening tools should:

1. employ a representative or unselected general sample of pregnant and/or postpartum people;
2. assess screening tool(s) against gold standard diagnostic assessments (for mental health conditions, semi-structured diagnostic interview);
3. assess, minimally, the core AD with or without OCD and PTSD;
4. ensure diagnostic assessments are conducted blind to screening test(s) results;
5. ensure appropriate timing between screening and diagnostic assessment; and
6. power to adequately detect the above metrics.

Beyond the above methodological criteria needed to correctly assess screening tool accuracy, studies assessing screening tool accuracy must also report screening tool metrics sufficient to allow an assessment of accuracy. Specifically, studies should measure diagnostic accuracy, reporting minimally, area under the curve (AUC), and sensitivity and specificity at specified cut-offs (both with 95% Confidence Limits). The AUC, sensitivity and specificity should also be reported independently of other disorders (e.g., depression). To merit broad dissemination, screening tool metrics should meet a certain threshold of accuracy. In our opinion, the empirical literature supports the following minimum criteria be met for a screening tool to be deemed “sufficiently accurate” as to merit implementation [68–70]:

- An AUC of ≥ 0.8 (≥ 0.8 is generally considered excellent) [71].
- A Youden’s “*J*” index (Youden 1950) of ≥ 0.5 (i.e., when sensitivity = 0.75, specificity ≥ 0.75).
- A negative predictive value (NPV) ≥ 0.8.
- A positive likelihood ratio (LR +) of ≥ 4.0. A LR + of 4.0 means that with a positive test result, the probability the person has the disease increases 25% over pre-test probability [72].

The overwhelming consensus from the perinatal AD screening literature, and experts in the field, is that the evidence meets neither: (a) the necessary methodological criteria described above, nor (b) the threshold of “sufficiently accurate” outcome metrics. Consequently, the evidence base remains inadequate to enable clear recommendations regarding optimal screening practice [4, 6]. Although a large number of potential AD screening tools have been evaluated within perinatal populations and several reviews of this literature have been published [4, 6, 40, 73, 74], all published studies are hampered by one or more important methodological weakness. These include: failing to assess the full composite of the AD; reporting insufficient metrics [75–78]; reporting accuracy for depression and AD combined [75, 77, 78]; employing selected rather than representative or unselected samples (e.g., specific groups of perinatal people are excluded such as those with depression or medical risk in pregnancy) and administering diagnostic interviews prior to the administration of screening tools, influencing screening tool completion [79–84].

To our knowledge, only two studies of perinatal AD screening tool accuracy have been conducted using full gold standard methodology [5, 46, 85]. Data from one (reported across two publications) provides support for the Edinburgh Perinatal Depression Scale – Anxiety Items (EPDS-3A) and the Matthey Generic Mood Question (MGMQ) [5, 85]. However, insufficient information regarding screening tool accuracy was reported. Consequently, while this study can be used to support the inclusion of the EPDS-3-A and the MGMQ in future evaluations, it cannot be used to draw firm conclusions regarding their accuracy. Further, our own study of postpartum people, some, albeit weak, support for the EPDS-3-A and the General Anxiety Disorder – 7 (GAD-7) was found [46].

Although existing studies’ findings do help to select tools deserving of further investigation, they fall far short of identifying an accurate and reliable, much less an optimal, screening tool for perinatal AD. Despite an urgent public health need, to date, no accurate perinatal AD screening tools have been identified. The proposed research aims to fill this gap.

### Objectives

The core objective of this research is to identify one or more accurate screening tools to detect perinatal AD. To this end, we will:

1. Assess the accuracy of a range of screening tools for perinatal AD as a whole and individually. Specifically, we will report the full composite of screening tool metrics for each evaluated measure:
  i) at each assessment point (prenatal and postpartum);
  ii) for the core DSM-5 anxiety disorders alone, and including OCD and PTSD;
  iii) for each of the individual AD; and
  iv) across parity (parity = 0, parity ≥ 1) and major ethnic groups.
2. Based on the above, we will report the most accurate screening tool for the AD as a group, and for each individual AD.

Secondary and exploratory study objectives:

1. Document the prevalence of perinatal AD in pregnancy and the postpartum. As this study requires an unselected/representative sample of perinatal people from within a specified geographical region, it provides the opportunity for us to document prevalence.
2. Explore relationships between participant demographic characteristics (e.g., age, relationship status, race/ethnicity, household income, income needs, education, employment status, geographical area of residence), identity (gender, sexual orientation), experiences with discrimination (e.g., race, social class, religion, weight/body size, chronic illness, history of substance use), and sleep with AD.
3. Assess perinatal people’s experience with mental health assessment and treatment seeking and utilization. Among participants who reported symptoms meeting criteria for one or more AD, we will assess assessment and treatment seeking as well as any barriers experienced (e.g., cost, location, race or culture, time, etc.). Among those who received an assessment or treatment, we will ask about the nature, duration, and costs associated with any assessments or treatment received, as well as diagnoses obtained, and practitioner type.
4. Assess the relationship between unpaid domestic labour (i.e., household chores and childcare responsibilities) and mental health and relationship satisfaction. Specifically, we will assess the overall burden (i.e., quantity), perceptions of fairness, and gender distribution of unpaid domestic labour with respect to mental health and relationship satisfaction prenatally and postpartum.

## Methods

### Study design

The current study employs a prospective cohort design.

### Inclusion criteria

All pregnant individuals, living in the province of British Columbia (BC), over the age of 18 and able to speak and read English at a level sufficient to complete the study questionnaires and interviews are eligible to participate in this research.

### Recruitment

We are employing a range of recruitment approaches with an emphasis on internet-based strategies, specifically, paid social media advertisements utilized for general population recruitment throughout the province. This method is supplemented with unpaid internet-based community recruitment to target rural, diverse, and marginalized communities. This entails the posting of study advertisements and posters on our lab social media sites (e.g., Facebook, Instagram, Twitter) and other online settings frequented by pregnant people in BC (e.g., pregnancy-related blogs, Facebook or Instagram pages, and websites).

Diverse sample recruitment is being carried out via partnerships with midwifery clinics, family physicians’ and obstetricians’ offices, and community centres and other community organizations. This recruitment consists of an introductory phone call or email to the agency, followed by an email including study information, study pamphlet, and posters to print or share with clients and the community. This form of recruitment is used to focus efforts on collecting a diverse, proportionally representative sample of the birthing population in the province and allows us to target additional populations that would otherwise not be reached through social media.

### Sample representativeness

To achieve a sampling frame that is 80–90% representative of the population of English-speaking birthing people in BC, participants are being recruited proportionately across BC’s five Health Authorities. To further enhance sample representativeness, sample characteristics (e.g., age, parity, education, geographical area) are tracked and compared to the BC perinatal database population data for the timeframe of recruitment. As needed, enhanced recruitment and sample weighting will be used to adjust for differences. All recruitment materials are inclusive of minorities and gender-diverse people.

### Sample size estimation

Approximately 44,000 residents of BC give birth each year [86]. We are seeking a sample of 1,000 participants for each of the two study time points. This will provide 80% power to detect AUC for ROC curves ≥ 0.56 as significantly different from 0.5, at a significance level = 0.05; 88% power to detect sensitivity ≥ 0.60 as > than 0.5; and > 99% power to detect specificity ≥ 0.60 as > 0.5. We will be able to estimate the prevalence of low prevalence conditions (e.g., 3%) with 1.5% margin of error and 95% confidence. Of note, we will have 80% power to detect AUC ≥ 0.65; 80% power to detect sensitivity ≥ 0.72; and 99% power to detect specificity ≥ 0.72. Allowing for an anticipated attrition of 15%, we are seeking to recruit 1,200 participants. To have 80% power to detect sensitivity ≥ 0.7; and 99% power to detect specificity ≥ 0.6 for the individual AD, we will need ≥ 30 participants per AD in this sample.

### Ethics

#### Ethics approvals

Approval for this study has been granted by the University of British Columbia’s Behavioural Research Ethics Board (#H20-01277).

#### Ethical and safety considerations

At the start and end of each questionnaire, all participants are provided with a list of pregnancy and postpartum-related resources in their community. During study interviews, any participant who reports clinically significant symptoms, or symptoms meeting diagnostic criteria for one or more AD, are provided a list of treatment services in their community, as well as individualized referral suggestions from the study interviewer.

If it is determined that a participant is suffering from suicidal ideation, additional steps are taken to ensure participant safety. If a participant endorses suicidal ideation during the completion of the online questionnaire (i.e., answers ‘Yes, quite often,’ or ‘Sometimes,’ to: “The thought of harming myself has occurred to me.”) on the measure administered to assess depressed mood (i.e., the Edinburgh Perinatal Depression Scale) [43], an additional risk assessment questionnaire then probes for suicidal behaviour. If a participant is deemed to have elevated risk for suicidal behaviour, the study team is alerted, and the participant is contacted via phone by a trained team member within 24 hours. If no contact is made, a follow up email is sent to maximize earliest participant contact. If suicidal ideation is identified during a study interview, the interviewer takes the necessary steps to ensure the participant’s safety. Management of suicidal ideation is based on the study safety and emergency mental health protocol.

In the event of a perinatal loss, participants are contacted by a member of the study team to express support, and perinatal loss specific resources are provided if desired with the option for additional support or resources at a later time, if needed. Only data provided prior to a perinatal loss will be retained in the data set (e.g., in the event that a loss occurs following the completion of the prenatal questionnaires and interview, data will be retained).

### Data collection and storage

Study questionnaires are completed online via REDCap [87, 88], and study interviews are administered by telephone or Zoom video conferencing [89].

#### Consent

Consent for this project is provided electronically at the time of enrollment. Interested individuals are able to access the study intake form from advertisements and are able to follow links to the consent form and eligibility questionnaire. Upon providing consent, participants are then able to download and save a PDF copy of the consent form for their personal records. By consenting to participate, participants are also consenting to complete the study interviews. Participants are informed of limits to confidentiality (i.e., urgent risk of harm to oneself, or harm coming to a child) in the consent form and at the beginning of each interview.

Consent for recording the interviews (for the purpose of assessing inter-rater reliability) is requested verbally at the beginning of each interview.

#### Participant communication

Personal, identifying information (e.g., name, first three digits of postal code, telephone) is acquired via the study intake form and stored in REDCap. For the duration of participation in the study, first name and email address of the participants remained connected in REDCap along with a unique ID code in order to send participants questionnaire links, reminders, and other communication throughout participation in the study.

#### Protection of participant information

Once data collection is complete, all data will be downloaded and stored on a secure, password-protected University of Victoria computer server, and subsequently deleted from the REDCap server. At that time, all identifying participant information will be removed from the study data files, and data will be identified only by a unique ID code only. Participant contact information, along with ID codes, will be stored on a separate, password protected file to retain participant information for future contact and potential follow up surveys.

### Procedures

**Fig. 1:**
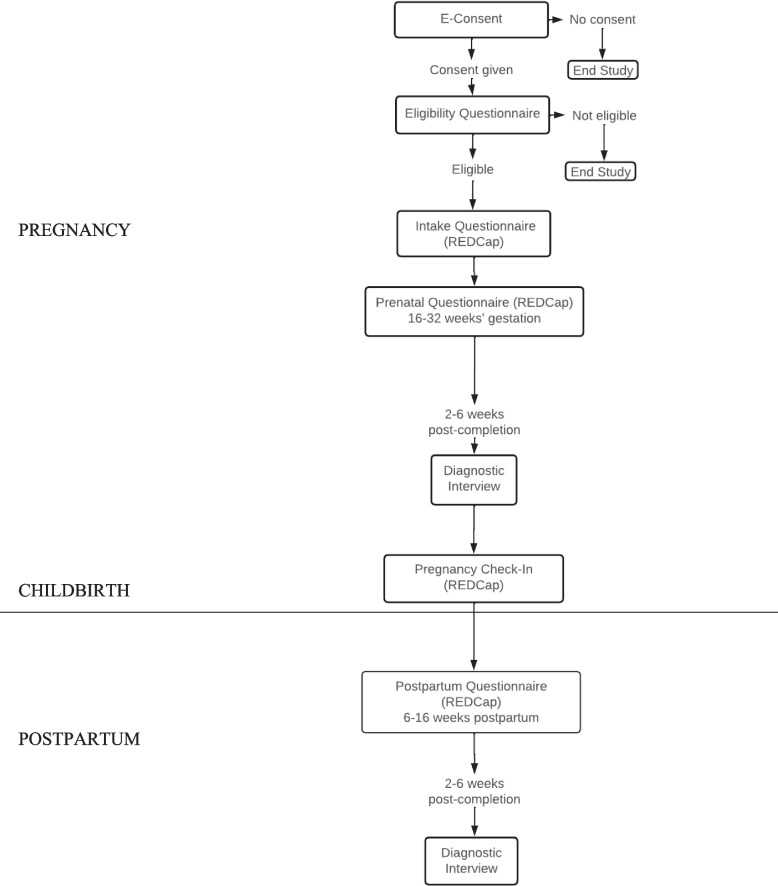
Participant flow

Following study enrollment, participants are administered questionnaires and an interview at two time points: once prenatally and once postpartum. Each assessment consists of online questionnaires and a semi-structured diagnostic interview. Participants are emailed a link to the questionnaires which they complete online. Prenatal questionnaires are completed between 16- and 32-weeks’ gestation. Postpartum questionnaires are completed between 6 and 16 weeks postpartum. All interviews are conducted over the phone or by video conference, 2–6 weeks after the completion of the questionnaires. See Fig. 1 for diagram of participant flow through the study.

### Assessment tools

See Table 1 for a summary of study measures.

**Table 1:** Summary of study measures

Domain	Measure	Method	Location
*Intake*			
Consent			Online
*Prenatal Questionnaire (16–32 weeks’ gestation)*			
Characteristics	Demographic and reproductive history information	Self-report	Online
AD Screening Measures	Perinatal Anxiety Screening Scale (PASS) State-Trait Anxiety Inventory (STAI-Y2) Edinburgh Postnatal Depression Scale (EPDS) Kessler Psychological Distress Scale (K6/K10) Matthey Generic Mood Questionnaire (MGMQ) Antenatal Risk Questionnaire (ANRQ-2A) Generalized Anxiety Disorder (GAD-7) Anxiety Disorders 22 (AD-22) Perinatal Anxiety Screening Questionnaire (PASQ)	Self-report	Online
Other functioning	Insomnia Severity Index (ISI) Covid-19 experience Family Work Quality Questionnaire (FWQQ)	Self-report	Online
*Prenatal Interview (2-4 weeks after prenatal questionnaire completion)*			
Diagnosis and onset (ADs, OCD, PTSD)	Diagnostic Assessment Research Tool (DART for DSM-5-TR)	Diagnostic Interview	Zoom or phone
*Pregnancy Check-In*			
Characteristics	Pregnancy status and birth date	Self-report	Online
*Postpartum Questionnaire (6–16 weeks postpartum)*			
Characteristics	Birthing information	Self-report	Online
AD Screening Measures	Perinatal Anxiety Screening Scale (PASS) State-Trait Anxiety Inventory (STAI-Y2) Edinburgh Postnatal Depression Scale (EPDS) Kessler Psychological Distress Scale (K6/K10) Matthey Generic Mood Questionnaire (MGMQ) Antenatal Risk Questionnaire (ANRQ-2A) Generalized Anxiety Disorder (GAD-7) Anxiety Disorders 22 (AD-22) Perinatal Anxiety Screening Questionnaire (PASQ)	Self-report	Online
Other functioning	Insomnia Severity Index (ISI) Covid-19 experience Family Work Quality Questionnaire (FWQQ) Mental health support seeking	Self-report	Online
*Postpartum* *Interview* *(2-4 weeks after prenatal questionnaire completion*)			
Diagnosis and onset (ADs, OCD, PTSD)	Diagnostic Assessment Research Tool (DART for DSM-5-TR)	Diagnostic Interview	Zoom or phone

#### Diagnostic interviews

##### **Diagnostic Assessment Research Tool (DART)** [90]

The DART is a psychometrically sound, semi-structured diagnostic interview designed to assess of a wide range of mental health conditions within the Diagnostic and Statistical Manual of Mental Disorders – 5th edition – Text Revision (DSM-5-TR) mental health conditions, including all the anxiety and anxiety-related disorders [90]. The DART includes mandatory, criterion-based questions and optional questions that may be necessary for clarification in order to make a diagnosis. Psychometric testing of the DART demonstrates excellent construct validity and good convergent and discriminant validity, and high interrater reliability [91]. The DART is used in this study to assess: (a) all DSM-5-TR anxiety disorders, (b) OCD, (c) PTSD, as well as (d) disorder age of onset and relation to reproduction. To assess perinatal AD specifically, the DART interview has been tailored to include perinatal specific examples (e.g., fear of childbirth, perinatal-related obsessions, birth trauma).

##### Interview training

Our team has extensive experience training students to administer diagnostic interviews to perinatal research participants. All study interviewers are trained to a strict criterion. Specifically, they are required to match the supervising interviewer: (a) on the primary diagnosis, and (b) to within ± 0.5 severity point, on two successive interviews to be permitted to interview independently. Study interviewers are also trained in special considerations in the assessment of perinatal AD, and the assessment of perinatal specific symptoms of the AD (e.g., worries about the baby, fear of childbirth). Study interviewers are closely supervised throughout the study.

#### Questionnaires

##### Anxiety disorder screening measures

Screening tools were selected based on two key criteria:

1. *Evidence of accuracy:* Measures which have been found in prior research, of reasonable methodological quality, to demonstrate some evidence of diagnostic accuracy (i.e., minimally an AUC ≥ . 70, and a Youden’s index of ≥ 0.4), have been included.
2. *Conceptually relevant:* Screening tools for which evidence of accuracy is not available but where the content is highly relevant have also been included.

To have the potential to be included based on screening accuracy, we required that the screening tool had been evaluated using sufficient quality methodology that the screening metrics could be deemed meaningful. Specifically, we required that the research assessed diagnostic accuracy in a perinatal population; employed some form of diagnostic assessment without knowledge of screening tool scores; assessed a minimum of three AD; and reported minimally AUC, and sensitivity and specificity for AD independently of other disorders. This represents a “softer” set of criteria than the full gold standard criteria, allowing us to determine which perinatal AD screening tools hold promise and should be further evaluated. Further, reported AUC values and *J*-Indices were required to meet or exceed 0.75, and 0.40, respectively.

Based on these criteria, we identified eight measures for inclusion in the current study: the STAI [92], the EPDS-3A [93], the GAD-7 [94], the K-10/K-6 [95], the MGMQ [5], the Perinatal Anxiety Screening Scale (PASS) [96], and the ANRQ-2A [97, 98]. Additional measures were assessed but performed too poorly to merit further evaluation: the KMMS [82] the EPDS [43], the GAD-2 [94], the Hospital Anxiety and Depression Scale – Anxiety (HADS-A) [99], the Pregnancy-Related Anxiety Questionnaire – Revised (PRAQ-R/R2) [100], and the Whooley [101]. Additional information about each measure is provided below.

State Trait Anxiety Inventory—Trait (STAI-T) [102]

The STAI-T is a 20-item self-report measure. Participants are asked to indicate how they feel in general on a 4-point Likert scale ranging from 0 (not at all) to 4 (very much so), in response to a variety of statement (e.g., “I have been nervous”). Scores less than 25 represent little to no anxiety, scores ranging from 26–40 represent moderate anxiety, scores over 40 represent high anxiety. The STAI-T has been used to assess clinical anxiety across many patient populations with a high Cronbach’s alpha of 0.90 [102]. The STAI has shown acceptable test–retest across samples (0.73-0.86) [102].

We elected to include the STAI-T was based on the single study of *N* = 100 pregnant people [79]. In this study, 5 of 8 AD were assessed, and both the AUC value (0.89) and the *J*-index (0.61) were above the threshold for a “good enough” screening tool.

##### **Edinburgh Postnatal Depression Scale – 3 Anxiety Items (EPDS-3A)** [43]

The EPDS is a 10-item self-report measure screening tool, and is the most widely used screening tool for postpartum depression [43]. Items are rated from 0–3 and assess symptom severity over the last week, with 0 indicating no symptoms, and 3 indicating more severe symptoms of depression. All 10 items are scored to provide a sum score, with higher scores indicative of more severe depressive symptoms. The EPDS has been included in the current study for two reasons: (a) to screen for depressed mood, and (b) to be evaluated as a screening tool for perinatal AD.

##### Screening for depression

A cut-off score of 13 identifies pregnant and postpartum people with severe depressive symptoms that should be assessed further by healthcare practitioners for a depression diagnosis (42). A review paper of the EPDS reported sensitivity estimates ranging from 64 to 100% for identifying depression, and specificity range from 73 to 97% across included studies (*n* = 11) [103]. Although the EPDS has presented with variable levels of sensitivity and specificity in the literature, it is a widely used tool in English-studies [104], and has also been highly acceptable across different cultures [103]. The EPDS has acceptable reliability and internal consistency (Cronbach alphas from 0.82—0.84) [105], and is a common screening tool for depression during both pregnancy and the postpartum [103, 105].

##### Screening for perinatal AD

Three items have been frequently found to cluster on an anxiety factor leading to the creation of an anxiety subscale, the EPDS-3A, which can be used to screen for perinatal anxiety [93, 106]. Studies have found the optimal cut-off score for anxiety to be 4 or above [107], or 6 or above [108]. When a cut-off of 4 has been used, the EPDS-3A has resulted in a sensitivity of 63%, and specificity of 70% for detecting any anxiety disorder [107]. Importantly, it has been found that women who score high on the anxiety subscale tend to score relatively low on the total EPDS, indicating that their anxiety may have gone undetected had the EPDS-3A was not specifically used [108].

To our knowledge, a total of five studies have assessed the accuracy of the EPDS-3A as a screening tool for perinatal AD [5, 46, 77, 80, 109]. Of these, the measure demonstrated sufficient accuracy as to provide support for inclusion in the current research [5, 46, 98]. In two of these studies, the EPDS-3A was assessed among pregnant people [5, 98]. In the first of these three (5), the screening accuracy of the EPDS-3A was assessed for six AD among *N* = 214 pregnant participants. In the second study [98], four AD were assessed in a sample of *N* = 954 pregnant people at approximately 34-weeks’ gestation. The third study [46] to provide evidence of the screening accuracy of the EPDS-3A included assessments of all the AD, and involved *N* = 310 postpartum participants. All three studies employed DSM-IV diagnostic criteria. The two studies involving pregnant people resulted in AUCs of 0.82 and 0.81, and *J*-indices 0.42 and 0.54, respectively [5, 100]. The study involving postpartum people [46] resulted in an AUC of 0.76, and a *J*-index of 0.42. Neither of the remaining two studies to evaluate the EPDS-3A as a screening tool for perinatal AD [80, 109] resulted in screening metrics meeting required thresholds.

##### **Generalized Anxiety Disorder 7-item Scale (GAD-7)** [110]

The GAD-7 is a 7-item self-report measure designed to assess symptoms of generalized anxiety disorder (GAD) [110]. Items are rated on a Likert-type scale, ranging from 0 (not at all), to 3 (nearly every day). The GAD-7 has been found to demonstrate good reliability as well as convergent, criterion, construct, factorial and procedural validity [110, 111]. The GAD-7 is sensitive to change over time [111], and has been validated for use in the perinatal period [77]. Prior research has found a Cronbach's alpha of 0.92 for the GAD-7 [110]. The GAD-2 is a shortened version of the GAD-7 comprised of the first two items, with these items selected to capture the core diagnostic features of GAD [94].

Inclusion of this measure was based on two studies: one previously conducted by our team (*N* = 310) [46], and a second more recent publication (*N* = 954) [98]. In the first of these [46], we assessed the screening accuracy of the GAD-7 for all AD as a group, against DSM-IV criteria, among postpartum people. This resulted in an AUC of 0.78 and a *J*-index of 0.41, exceeding our threshold for inclusion. The second study’s [98] assessment of the screening accuracy of the GAD-7 was conducted in relation to five DSM-V AD (i.e., GAD, PD, AG, SAD and OCD) criteria. This resulted in an AUC of 0.82 and a *J*-Index of 0.51, also exceeding our threshold for inclusion. In the same study [98], the GAD-2 was also assessed for these disorders and resulted in even stronger screening metrics (i.e., an AUC of 0.83 and a *J*-Index of 0.52).

##### **Kessler Psychological Distress Scale (K6/K10)** [95]

The K10 is a self-report measure of psychological distress which can be administered as the full 10-item version (the K10) or the briefer, 6-item version (the K-6) [95]. The K6 is comprised of 6 items from the K10. Items are measured on a 5-point Likert scale ranging from 0 (none of the time), to 4, (all of the time). Total scores range from 0–24 for the K6, and 0–40 for the K10. Optimal cut-scores for the detection of DMS-IV mental health conditions have been suggested as 14 for the K6, and 24 for the K10 [112]. The K10/K6 has demonstrated consistently strong psychometric properties across a variety of sociodemographic samples [95], and the K6 has been found to have result in a sensitivity of 0.36 and a specificity of 0.96 in predicting serious mental illness in the general population [113].

The K10/K6 as a screening tool for perinatal AD has been evaluated in two studies [80, 81]. This resulted in two assessments of the K10 [80, 81], and one of the K6 [80]. The first assessment of the K10 as a screening tool for perinatal AD employed a representative sample of pregnant people (*N* = 129), and assessed 3 AD [81]. This evaluation resulted in AUC values of 0.71 and 0.76, and *J*-Indices of 0.48 and 0.75 (depending on the cut-score employed) [81]. The second study to evaluate the K10 as a screening tool for perinatal AD also employed a representative sample of pregnant people (*N* = 376), and included an assessment of all of the AD, resulting in an AUC of 0.77 and a *J*-Index of 0.46 [80]. This second study also included an assessment of the K6 which resulted in an AUC of 0.77 and a *J*-Index of 0.45.

##### **Matthey Generic Mood Question (MGMQ)** [5]

The MGMQ screens for a range of negative emotional states including depression and anxiety [5]. It consists of three questions pertaining to how the individual has been feeling over the past two weeks [5]. The first question (distress question) is related to feeling stressed, anxious, or unhappy (response options of ‘yes’, ‘possibly’ or ‘no’). If this initial question is endorsed, the individual is asked the two remaining questions: how bothered (bother question) they have been by these feelings (response options of ‘not at all’, ‘a little bit’, ‘moderately’, ‘a lot’), and what they think has caused their distress. Although the MGMQ appears to demonstrate a good–excellent level of accuracy in screening for perinatal anxiety disorders [114] publications related to this measure have not included metrics necessary to draw this conclusion with confidence [5], nor have they consistently differentiated screening accuracy for depression from screening accuracy for anxiety [85].

##### **Perinatal Anxiety Screening Scale (PASS) ** [96]

The PASS [96] is a 31-item self-report measure designed to screen for perinatal anxiety. Participants are asked to indicate how often they have experienced a variety of emotions/sensations (e.g., worry about the future) over the last two weeks. Items are scored via a 4-point Likert scale ranging from 0 (not at all), to 4 (almost always). Scores in the 0–20 range represent little to no anxiety, scores ranging from 21–41 represent mild-moderate anxiety, and scores ranging from 42–91 represent severe anxiety.

The inclusion of the PASS in the current study was based on a study in which the PASS, when administered to pregnant participants (*N* = 312), demonstrated very high accuracy against both DSM-IV and the ICD-10 diagnostic criteria (i.e., AUC values of 0.93 and 0.94, and *J*-indices of 0.76 and 0.79, respectively) [83]. However, the strong screening metrics obtained in this study may be attributable to the fact that study interviews were administered prior to questionnaire completion. Administration of the study interview prior to questionnaire completion may have resulted in participants providing responses more similar to those provided in the interview than would have been the case had the questionnaire been completed first.

##### **Antenatal Risk Questionnaire – 2 Anxiety Items (ANRQ-2A) ** [97]

The ANRQ is a 12-item questionnaire that assesses a range of content domains (e.g., emotional support from respondent’s own mother in childhood, history of mood disorder and treatment received, life stress) [97]. The ANRQ-2A is comprised of the two anxiety items from this measure, with higher scores indicating risk for perinatal mental health problems [95]. ANRQ scores have been shown to correlate with EPDS scores [115].

The ANRQ-2A was included based on a recent study of *N* = 954 people assessed in late pregnancy [98]. In this study, the accuracy of ANRQ-2A was assessed against DSM-IV diagnostic criteria. A cut-off of 6.0 on the ANRQ-2A resulted in an AUC of 0.84, and yielded a *J*-Index of 0.54 (in this case both sensitivity and specificity = 0.77), providing support for these items in routine screening [98].

##### **Anxiety Disorder—22 (AD-22) ** [116]

The AD-22 is a 22-item, self-report anxiety screening measure created for the current project [116]. Instructions, items, and scoring was adapted from a previous proof of principle screening accuracy assessment of 13 items (1–3 per disorder) taken from a variety of other self-report measure of specific anxiety conditions. The AD-22 measure includes items reflective of each of the core AD (i.e., PD, SAD, SP, GAD, AG, separation anxiety) as well as OCD and PTSD. Items were developed by our team of investigators. There are three items for each disorder with the exception of SP and AG for which there are two, respectively. Respondents are asked how bothered they have been by each of the 22 included symptoms/experiences of anxiety (e.g., difficulty controlling worry, feeling afraid of crowded spaces) over the previous two weeks. Items are rated on a Likert-type scale from 0 (not at all) to 4 (extremely).

This measure has been included in the current study as the 13-item version exceeded the required accuracy of a “good enough” screening tool [46]. Specifically, data from this study of N = 310 postpartum people resulted in an AUC of 0.86 and a *J*-index of 0.54.

##### **Perinatal Anxiety Screening Questionnaire (PASQ)** [117]

The PASQ is a newly constructed screening tool, created by our study team specifically for perinatal AD screening [117]. The PASQ screens for all core AD (PD, SAD, SP, GAD, AG, separation anxiety), as well as OCD and PTSD. The tool includes 8 primary “yes/no” questions to probe for the core feature of each AD (e.g., “Do you worry a lot about many different things [e.g., your pregnancy or baby, parenting ability, family finances, work or school, or world events]?”). For each AD, if the participant selects “yes” to the primary question, the next question for that AD section is asked. However, if the participant selects “no” to the primary question, they will move to the next AD section and will be asked a new “yes/no” primary question for a new AD. The PASQ has a total of 36 possible questions, and the number of questions for each AD section varies depending on the number needed to effectively address the core DSM-5 diagnostic criteria of each AD. There are 3 possible questions for SP, 6 for PTSD, 7 for OCD and the remaining AD sections (SAD, PD, AG, GAD, separation anxiety) all have 4 possible questions each. This tool was created for the current project to include examples and descriptions catered to new and expectant parents.

#### Additional measures

##### Insomnia Severity Index (ISI)

The ISI is a 7-item, self-report measure of sleep difficulties experienced over the previous two weeks [118]. Items are rated on 0–4, Likert-type scales with 0 representing an absence of symptoms, and 4 representing severe symptoms. Items are summed to yield a total score, with higher scores representing more severe sleep difficulties. Scores between 15–21 indicate moderate insomnia, while scores over 22 indicate severe insomnia. In clinical samples, a cut-off of 15 has yielded a sensitivity of 78% and a specificity of 1.00 for detecting cases of insomnia [119]. Evidence of ISI validity has been demonstrated by moderate but significant correlations between ISI scores and both subjective (sleep diary) and objective (polysomnography) measures of sleep [119]. Similarly, the ISI has shown sustained sensitivity to changes in sleep difficulties over time, with total scores being paralleled by sleep diary data, polysomnography and clinician evaluation [118]. This measure has shown excellent internal consistency in clinical and community samples (Cronbach alphas from 0.73—0.91), and has been deemed a reliable tool to measure insomnia severity [118, 119].

##### Family Work Quality Questionnaire (FWQQ)

The FWQQ, developed by Janzen and Hellsten [120] is a 28-item self-report measure of unpaid family work. This measure assesses five subscales of unpaid work quality: demands (time pressure and differing demands); equity (fairness in the division of unpaid work); autonomy (freedom over types of work conducted and decision); social resources (assistance from family and friends); caregiving reward (gratification received from responsibilities). Items within each subscale are rated on a 4-point Likert scale from 1 (strongly disagree), to 4 (strongly agree). The scale has shown adequate internal consistency, with Cronbach alphas ranging from 0.75-0.88 [120].

##### Lab based measures

Questions pertaining to participant demographic information, identity, experiences, reproductive history, current pregnancy and birth, childcare distribution, relationship and sexual satisfaction, postpartum mental health service utilization and covid-19 were developed by our research team and included in the study questionnaires. Much of this content has been used before in previous studies [46, 121, 122].

##### Demographic information

Our team has well-established questions pertaining to participant demographic information which we have used in multiple studies [46, 121, 122]. Demographic characteristics assessed include age, relationship status, race/ethnicity, household income, income needs, education, employment status, geographical area of residence.

##### Identity

Questions to better understand participants’ identity, including sex assigned at birth, gender, pronouns, sexual orientation, belonging to a minority community have been included. Additionally, participants are asked if they have experienced discrimination during their medical care due to their identity or an experience (e.g., race, social class, religion, weight/body size, chronic illness, history of substance use).

##### Reproductive, pregnancy and birthing information

Our team has well-established questions pertaining to participant reproductive history, current/most recent pregnancy, and birth [46, 121, 122]. Information collected includes parity, delivery, number of children (biological, non-biological), reproductive loss, conception, birth plan, mode and location of delivery, birth weight, neonatal health, infant feeding, infant sleep arrangement.

##### Domestic labour

We are also collecting information pertaining to domestic labour. Questions ask about the participant’s parenting/living situation, parental leave, childcare, share of care of children and share of domestic responsibilities.

##### Satisfaction

Relationship satisfaction, sexual satisfaction, and irritation and/or anger with the division of childcare and domestic chores were each asked about via a single question. Relationship satisfaction was rated on a 4-point Likert scale ranging from “Very dissatisfied” to “Very satisfied.” For sexual satisfaction, participants are asked to select a response ranging from “Dissatisfied” to “Satisfied,” with an additional option to select “I choose not to answer.” The irritation and/or anger with the division of childcare and domestic chores was rated on a 5-point Likert scale ranging from “Not at all” to “Extremely.”

##### Postpartum mental health

In the postpartum questionnaire, participants are asked to provide information regarding their engagement with mental health services. Specifically, they are asked if they have sought any mental health assessment(s) since their prenatal interview with us. If an assessment for mental health difficulties was sought, they are then asked if they experienced any barriers (e.g., cost, location, race or culture, time, etc.). If they received an assessment, they are asked additional questions about diagnosis and treatment (e.g., which, if any diagnoses were assigned, what, if any, treatment(s) they are receiving, how this treatment is funded and what practitioner provides this treatment if applicable).

##### COVID-19

Nine questions created by our research team regarding COVID-19 are included to assess the impact of the pandemic on participants’ experience of anxiety. Participants are asked questions relating to their experience and concerns related to COVID-19 (e.g., “How concerned are you about the potential impact of COVID-19 on your childbirth experience?”; “How much of an impact is COVID-19 currently having on your ability to receive support care for your children?”). Participants are asked to select a response ranging from “Not at all” to “Extremely.”

### Current status

Recruitment, data collection and study interviews are currently underway and are set to finish in January 2025. To date, we have recruited approximately 60% of our sample, with 783 participants enrolled. Of the 727 enrolled, 316 of these have fully completed their participation in the study.

### Data analysis plan

#### Descriptive statistics

Descriptive information will be presented as means, standard deviations, percentages, and 95% confidence intervals. The diagnostic accuracy of screening tools will be assessed via receiver operating characteristic (ROC) curve analyses within the prevalence sample. AUC values, derived from the ROC curves, represent overall diagnostic accuracy, and will be reported with 95% confidence intervals. Optimal cut-off values for each measure will be determined from ROC analyses and based on maximizing Youden’s *J* index value (see screening tool metrics above). The sensitivity, specificity, PPV and NPV, LR ± , and percent correct classification will also be reported. We will also determine the cut-point associated with 0.75 sensitivity and calculate the corresponding *J* index and specificity (prevalence sample). We will estimate sensitivity and specificity of each of the screening tools for the individual AD.

#### Research objectives

AUC will be compared among all pairwise combinations of tools using DeLong’s method for paired ROC curves [123] and using a false-discovery-rate correction (FDR, with family-wise alpha = 0.05) for *p*-values [124] to account for multiple testing. Sensitivity and specificity will be compared across all tools using McNemar’s test for paired categorical data, with FDR correction for multiple testing [124].

We will report the full composite of the screening tool metrics (i.e., AUC, *J* index, PPV, NPV, LR + , and LR-) for each of the measures assessed. Screening metrics will be reported for the core AD with and without PTSD and OCD, as well as separately for major ethnic and parity groups (i.e., parity = 0, and parity ≥ 1). We will recommend screening tools based on their accuracy (i.e., scores on the above metrics) and implementation feasibility (i.e., length, administration time, scoring time and complexity, and cost, if any). To ensure the screening tools are not missing any individual AD by analyzing them together, we will also estimate sensitivity and specificity for each AD by comparing those who report symptoms meeting criteria for one specific AD with those whose symptoms fail to meet criteria for any AD.

#### Additional analysis

We will use log-binomial models to evaluate the relative risk of AD with respect to important demographic and reproductive variables (e.g., age, and parity), COVID-19 related concerns, and gender role burden (e.g., time spent in childcare and domestic tasks). We will evaluate these variables as risk factors for AD development and exacerbation. In the unlikely event that none of the assessed screening tools meets the standard of a sufficiently accurate measure, data from the first assessment will be used to empirically derive a new measure. This would involve exploratory and confirmatory factor analysis, followed by logistic regression and prediction modelling, using the screening tool items from the first assessment as training, and the second assessment as testing and validation.

#### Software

Data management and analyses described above will be conducted using R [125] and Statistical Package for Social Science (SPSS) (Version 25) [126].

## Discussion

It is now evident that perinatal AD are highly prevalent (1 in 5 perinatal people), impairing, distressing with potential negative effects for the developing fetus and infant [15–17]. Detection, prevention, and treatment are highly important to mitigating these consequences. Accurate and reliable screening is a critical first step to identifying those at risk of, or currently suffering from, these disorders. Despite calls for implementation [62, 64], perinatal AD screening is rarely conducted. This is in large part because the current published literature fails to provide sufficient empirical support for any instrument. Although much research evaluating perinatal AD screening tools has been conducted, it has been hampered by methodological shortcomings of such magnitude that no available screening tool can justifiably be recommended for widespread implementation.

For these reasons we have undertaken a large-scale study of the accuracy of a broad range of potential perinatal AD screening tools. Our objective is to identify, employing gold standard methodology, one or more accurate and reliable measures. We have sought to overcome the methodological shortcomings of previous research so that our findings can be justifiably used to make practice and policy decisions. Population-based administration of screening tools is expensive to implement and is unlikely to be undertaken without strong evidence that the screening tool(s) employed will in fact correctly identify those experiencing the disorders being screened for.

To our knowledge, this will be the first study in which all gold standard methodology has been employed. Specifically, in this research:

- all of the core anxiety disorders and additional anxiety-related disorders are assessed;
- gold standard, diagnostic interviews are administered;
- diagnostic assessments are conducted blind to screening tool results;
- DSM-5 diagnostic criteria are employed;
- an unselected/representative sample of pregnant and postpartum people is being sought;
- screening accuracy is assessed in both pregnancy and the postpartum; and
- diagnostic interviews are administered within two to six weeks of completion of screening tools.

The study includes a number of additional methodological strengths. Specifically, the study is powered to ensure we can assess the accuracy of included screening tools both for the AD as a whole, but also for each individual AD. Our approach to recruitment is designed to result in data for 1,000 pregnant and 1,000 postpartum people. We are over-sampling, where needed, perinatal people with specific AD so that we are able to report on screening accuracy for each of the individual AD.

Beyond the core goal of this research (i.e., to assess the accuracy of various self-report measures when used as screening tools for perinatal AD), we have the opportunity to explore a number of additional research questions. For example, because this research is based on DSM-5 anxiety and related disorders, we are also able to explore screening for separation anxiety disorder. To our knowledge, no findings related to screening for adult separation anxiety among perinatal people have been published. In addition to providing information about screening accuracy for adult separation anxiety disorder, we will also be able to provide the first estimate of the prevalence and incidence of this condition among perinatal people. Further, we will document the point prevalence and postpartum incidence of perinatal AD in pregnancy and the postpartum. To our knowledge, perinatal AD prevalence, based on DSM-5 criteria has yet to be reported. Further, we will be able to assess the extent to which participants who report symptoms meeting criteria for one or more AD go on to receive an assessment and treatment for these conditions. We will also assess various predictors (e.g., demographic, reproductive, identity, experience of discrimination) of perinatal AD symptoms and diagnosis, as well as assessment and treatment access and utilization. Finally, we will assess the relationship between the burden and gender distribution of unpaid domestic labour (i.e., household chores and childcare responsibilities) and mental health.

Findings from this research will provide rich data to answer not only our core research questions but additional, important questions pertaining to perinatal AD.

## Data Availability

The datasets generated and/or analysed during the study will be available from the corresponding author upon reasonable request following the completion of all primary publications derived from these datasets.

## Abbreviations

AD: Anxiety and anxiety-related disorders
AG: Agoraphobia
AUC: Area under the curve
BC: British Columbia
COPE: Australian clinical practice
DSM-5: Diagnostic and statistical manual of mental disorders
LR: Likelihood ratio
NPV: Negative predictive value
OCD: Obsessive–compulsive disorder
PPV: Positive predictive value
PTSD: Posttraumatic stress disorder
PD: Panic disorder
ROC: Receiver operating characteristic
SP: Specific phobia
SAD: Social anxiety disorder
GAD: Generalized anxiety disorder
